# Multi-Feature Input Deep Forest for EEG-Based Emotion Recognition

**DOI:** 10.3389/fnbot.2020.617531

**Published:** 2021-01-11

**Authors:** Yinfeng Fang, Haiyang Yang, Xuguang Zhang, Han Liu, Bo Tao

**Affiliations:** ^1^School of Communication Engineering, Hangzhou Dianzi University, Hangzhou, China; ^2^College of Computer Science and Software Engineering, Shenzhen University, Shenzhen, China; ^3^State Key Lab of Digital Manufacturing Equipment & Technology, School of Mechanical Science and Engineering, Huazhong University of Science & Technology, Wuhan, China

**Keywords:** electroencephalogram (EEG), machine learning, feature exaction and selection, deep forest, emotion feelings-as-information

## Abstract

Due to the rapid development of human–computer interaction, affective computing has attracted more and more attention in recent years. In emotion recognition, Electroencephalogram (EEG) signals are easier to be recorded than other physiological experiments and are not easily camouflaged. Because of the high dimensional nature of EEG data and the diversity of human emotions, it is difficult to extract effective EEG features and recognize the emotion patterns. This paper proposes a multi-feature deep forest (MFDF) model to identify human emotions. The EEG signals are firstly divided into several EEG frequency bands and then extract the power spectral density (PSD) and differential entropy (DE) from each frequency band and the original signal as features. A five-class emotion model is used to mark five emotions, including neutral, angry, sad, happy, and pleasant. With either original features or dimension reduced features as input, the deep forest is constructed to classify the five emotions. These experiments are conducted on a public dataset for emotion analysis using physiological signals (DEAP). The experimental results are compared with traditional classifiers, including K Nearest Neighbors (KNN), Random Forest (RF), and Support Vector Machine (SVM). The MFDF achieves the average recognition accuracy of 71.05%, which is 3.40%, 8.54%, and 19.53% higher than RF, KNN, and SVM, respectively. Besides, the accuracies with the input of features after dimension reduction and raw EEG signal are only 51.30 and 26.71%, respectively. The result of this study shows that the method can effectively contribute to EEG-based emotion classification tasks.

## 1. Introduction

Emotions occupy a very important position in human communication and personal decision-making. Although the existence of emotion is well-known, human knows very little about the mechanism behind it. Traditionally, human–computer interaction (HCI) for emotion recognition is carried out by using voice and facial expression signals (Fan et al., 2003; Sidney et al., 2005; Zeng et al., 2008). But these external signals have a certain degree of camouflage. Using voice and facial expression signals as the basis for emotion recognition is therefore not convincing. EEG physiological signals are directly produced by the central nervous system of human body, and the central nervous system is closely related to human emotions. Zheng et al. (2017) proved that the neural characteristics and stable EEG patterns are related to positive, neutral, and negative emotions. It indirectly proves that the use of EEG signals for emotion recognition is reliable. Emotion recognition becomes a hot research topic regarding the development of basic emotional research theories and applications of emotional brain–computer interactions (aBCIs) (Nijboer et al., 2009; Garcia-Molina et al., 2013), such as emotion recognition with human brain-activity sensors is used for the treatment of patients with mental disorders (Mehmood et al., 2017).

In the field of EEG-based emotion recognition, traditional classifiers, such as K-Nearest Neighbor (KNN), Random Forest (RF), Gaussian Naive Bayes (GNB), Linear Discriminant Analysis (LDA), and Support Vector Machine (SVM), have been widely used. Heraz and Frasson (2007) used KNN to classify the intensity of each emotion (i.e., pleasure, arousal, and dominance) into two classes (high or low). With the database collected by themselves, the experimental accuracy reaches 73.5, 74.6, and 74%, respectively. He et al. (2017) proposed a feature extraction method based on multiple empirical mode decomposition (MEMD). The emotional state is identified as high/low arousal and high/low valence for the recorded eight-channel EEG signals on the DEAP database. The accuracy of SVM for arousal and valence is 67.9 and 70.9%, respectively. Veeramallu et al. (2019) utilized empirical mode decomposition (EMD) to classify automatic emotion classification based on EEG. This method uses the random forest classifier to classify positive, neutral, and negative emotions on the SJTU emotional EEG database (SEED) and obtains the highest recognition accuracy of 89.59, 91.45, and 93.87%, respectively. With the development of neural networks, deep learning based on neural networks and convolutional neural networks (CNN) have widely used in emotion recognition. Yang et al. (2018b) design a novel emotion recognition system which combines recurrence quantification analysis (RQA) with channel-frequency convolutional neural network (CFCNN). With the database collected by themselves, they classify the three specific emotions: happiness, sadness, and fear. The average recognition accuracy is 92.24%. Mehmood and Lee (2015) use KNN and SVM to classify four emotions: scared, sad, happy, and calm. With the database tested by themselves, the accuracy of emotion in valence and arousal dimensions is 32 and 37%, respectively by SVM, and the highest accuracy of KNN is 61%. Zheng et al. (2017) used discriminative Graph regularized Extreme Learning Machine (GELM) to perform LALV, HALV, LAHV, and HAHV (low arousal/low valence, high arousal/low valence, low arousal/high valence, and high arousal/high valence), four-classes classification experiments on valence-arousal (VA) space on the DEAP database, and achieve average recognition accuracy of 69.67%. The above results show that the accuracy of valence and arousal (two classes) have achieved good results. However, for the classification of 3 or 4 emotions, the recognition accuracy is generally not acceptable and needs to be improved. Therefore, how to improve the recognition accuracy of emotion classification for more classes and how to recognize the relationship between the generation of emotion and the corresponding physiological mechanism are the problems that need to be solved urgently in the field of BCI. This study aims to apply the deep forest technology to recognize human emotion from EEG signals, and improve the accuracy.

The layout of the paper is as follows. Section 2 introduces the development of multi-Grained Cascade Forest (gcForest) and its application in image classification and emotion recognition. In section 3, the DEAP dataset, the preprocess of EEG emotion recognition, the method of extracting features, the realization of the MFDF method, and the confirmation of hyper-parameters of deep forest are introduced. Section 4 demonstrates the experiment and experimental results, and section 5 analyzes and discusses the experimental results. Section 6 gives some conclusions and future work.

## 2. Related Work

GcForest is a highly competitive decision tree integration method for deep neural networks (Zhou and Feng, 2017). The gcForest consists of multi-grained scanning and cascade Forest. It employs a cascade structure to realize layer-by-layer processing. Through multi granularity scanning, they increase the diversity of features to enhance the cascade forest. In addition, the gcForest is a deep model based on decision trees, and the training process does not rely on back-propagation and gradient adjustment. Compared with deep neural networks, gcForest has fewer hyper-parameters and achieves excellent performance across various domains by using even the same parameter setting. In the study of Cao et al. (2019b), the rotation-based deep forest (RBDF) is proposed for the classification of hyper-spectral images (HSIs). Experimental results based on three HSIs demonstrate that the proposed method achieves the state-of-the-art classification performance. Cao et al. (2019a) propose a new deep model–densely connected deep random forest (DCDRF) to classify the HSIs. Experimental results prove that the proposed method can achieve a better classification performance than the conventional deep-learning-based methods. A deep multigrained cascade forest (dgcForest) was proposed by Liu et al. (2019). Experimental results testify that their proposed algorithm presents a good performance on the hyper-spectral image (HSI). Zhou et al. (2019) proposed a deep-forest-based method for hashing learning. The experimental results show that the proposed method has better performance with shorter binary codes than other corresponding hashing methods. In conclusion, in image detection, voice detection, and other fields, the gcForest has been applied and achieved excellent results.

Each cascade layer of gcForest is composed of random forest. Random forest is an algorithm that integrates multiple decision trees based on the idea of ensemble learning. Its basic unit is the decision tree, and these decision trees are independent of each other and have no relationship (Ho, 1995; Breiman, 2001). The integrated learning feature of random forest enables it to obtain better results even if each tree does not have high-precision decision-making. Random forest is utilized by Memar and Faradji (2017) to classify the sleep stage based on EEG signals, which is one of the most critical steps in effective diagnosis and treatment of sleep-related disorders. Random forest consists of decision trees. Decision tree is a shortcut mode of attribute classification (Janikow, 1998). Additionally, decision tree is a kind of white box method, and it is more convincing than other classifiers. In dealing with the undefined problem of emotion recognition, the results obtained by the decision tree help us understand the physiological mechanism behind the data that generates emotion. Since decision tree is proposed, it has been widely used in the treatment of diseases with EEG signals. According to previous studies, the induction of decision trees from data has been applied in various medical domains, and Hetmerova et al. (1997) argued that it is interesting to use decision tree to extract useful rules for disease judgments. For example, Rajaguru and Prabhakar (2017) proposed a soft decision tree classifier in the EEG seizure classification, and Sukanesh and Harikumar (2008) proposed hierarchical aggregation functions decision trees to classify epilepsy risk classification based on EEG signals. Based on the above studies, it is feasible to use gcForest based on decision tree for emotion recognition.

Multi-grained scanning is usually used to process the original data. For example, Cheng et al. (2020) propose a method for multi-channel EEG-based emotion recognition using deep forest. On the DEAP database, the average accuracy reaches 97.69 and 97.53% for valence and arousal, respectively. Yao et al. (2019) used deep forest with multi-scale window (MSWDF) to identify EEG emotions, and the average recognition accuracy in the classification of pleasure, relaxation, sadness (three classes) is 84.90%. These studies directly use multi-grained scanning on the original data to conduct experiments instead of using the feature extraction method. This paper proposes an emotion recognition algorithm model, the multi-feature deep forest (MFDF), on the basis of gcForest. The algorithm extracts effective features from the original data and inputs the features into the deep forest for emotion classification and recognition. The experimental results show that the average accuracy of MFDF reaches 71.05%, and the highest accuracy can reach 87.10% on the DEAP database. The experimental results prove our model is valid.

## 3. Method

**Figure 1:**
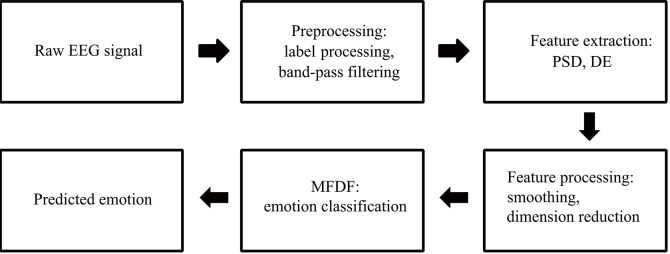
The flow chart of EEG-based emotion recognition.

The MFDF algorithm is shown in Figure 1. Firstly, the original data is preprocessed, including emotion label processing and frequency band division. Secondly, affective computing is performed on the data to obtain two types of features: PSD and DE. Thirdly, data smoothing and dimensionality reduction are performed on the features. Finally, the original data is converted into feature vectors, and the feature vectors are input to the deep forest for emotion recognition and classification.

### 3.1. Introduction to DEAP Dataset

The public dataset DEAP (Koelstra et al., 2011) is utilized to validate our proposed approach in this study. The dataset contains 32 channel EEG signals and eight-channel peripheral physiological signals recorded from 32 subjects watching 40 music videos. Each video is rated to the levels of arousal, valence, liking, and dominance of each subject. The rating scores are closely related to emotions. In the current study, only the EEG signals are used for emotional recognition. The EEG signals are sampled at the frequency of 128Hz, and then are preprocessed by a bandpass filter with a bandwidth ranging from 4.0 to 45.0 Hz. The recorded EEG data contains 60 s video-induced EEG data and 3 s baseline data without watching video. The EEG data from each subject are organized as shown in Table 1.

**Table 1:** Data organization for one subject.

**Identification**	**Size**	**Content**
Data	40 × 40 × 8,064	Video/trial × channel × data
Label	40 × 4	Video/trial × label (valence, arousal, dominance, liking)

### 3.2. Data Pre-processing

For the study of EEG signals, five frequency bands are normally separated with different frequency ranges: delta (1–4 Hz), theta (4–7 Hz), alpha (8–13 Hz), beta (13–30 Hz), and gamma (31–47 Hz). Except for the delta signal, which is generally generated during deep sleep, the theta, alpha, beta, and gamma signals are closely related to emotions (Aftanas et al., 2001; Balconi and Lucchiari, 2008; Balconi and Mazza, 2009); the theta, alpha, beta, and gamma signals are thus extracted in the current study.

This study marks angry, happy, sad, pleasant, and neutral according to the following rules.

$$\begin{array}{l} \text{Angry: Valence < 4.5 and Arousal > 5.5}\\ \text{Happy: Valence > 5.5 and Arousal > 5.5}\\ \text{ Sad: Valence < 4.5 and Arousal < 4.5}\\ \text{Pleasant: Valence > 5.5 and Arousal < 4.5 }\\ \text{Neutral: 4.5 }\leq \text{ Valence }\leq \text{ 5.5 and 4.5 }\leq \text{ Arousal }\leq \text{ 5.5} \end{array}$$

**Figure 2:**
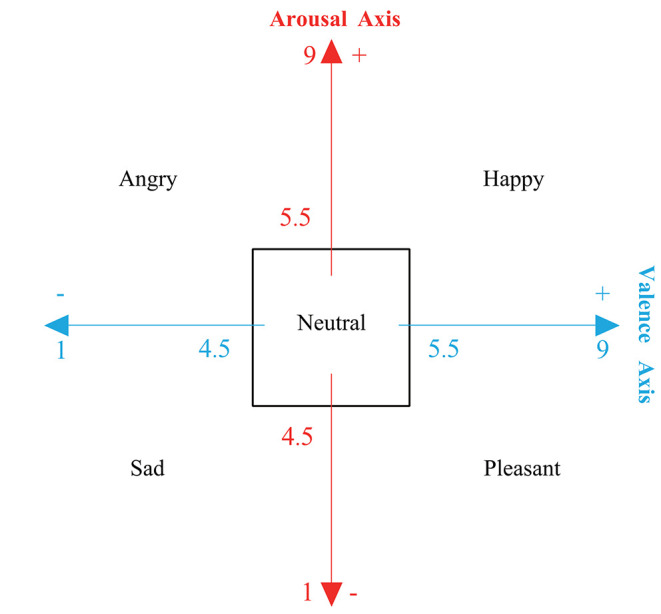
The emotion model for relabeling five emotions, including neutral, angry, happy, sad, and pleasant.

Related studies tend to classify the level of arousal and valence as two-class or three-class classification problems. Few studies provide emotional labels according to the scores of valence and arousal. Lan et al. (2016) mark the EEG signals with the labels of pleasant, happy, frightened, and angry. Zheng et al. (2017) proposed an emotion representation model based on the valence-arousal (VA) level and mark four quadrants of the VA space with four types of emotions. On the basis of these studies, the current study labels the data with five emotional classes, with an additional emotion type neutral, as shown in Figure 2. The data of subject S3, S12, S13, S14, S23, S26, S27, S30, and S31 are not used in this study for evaluation because some labels are absent after remarking by the proposed five-class emotion model.

### 3.3. Feature Extraction

Several features are extracted from the EEG signals for analysis, including power spectral density (PSD) and differential entropy (DE). Naderi and Mahdavi-Nasab (2010) found that the power spectral density (PSD) estimation of the Welch method can provide very strong features, and it is also a good representation of the EEG signal. DE has proven to be the most accurate and stable EEG feature that reflects the change of vigilance (Duan et al., 2013; Shi et al., 2013).

The PSD can be defined as follows,

(1) $$\begin{array}{l} P_{i}(f)=\frac{1}{L}|\sum_{n=1}^{L-1}{\text{x}}_{\text{i}}[\text{n}]{\text{e}}^{\text{j}2\pi \text{fn}}{\text{|}}^{2}, \end{array}$$

where *L* is the length of the signal, and *P*_*i*_(*f*) is the fast Fourier transform of the signal x_i_[n]. The PSD feature [*P*(x_i_)] of the signal x_i_[n] can then be obtained by Equation (2).

(2) $$\begin{array}{l} P({\text{x}}_{\text{i}})=\frac{1}{\text{K}}(\sum_{\text{i}=1}^{\text{K}}{\text{P}}_{i}(\text{f})), \end{array}$$

where, *K* is number of frequency points used to calculate the discrete Fourier transforms.

Feature DE can be defined as follows,

(3) $$\begin{array}{l} h({\text{x}}_{\text{i}})=-\int_{-\infty }^{\infty }\frac{1}{\sqrt{2\pi \sigma^{2}}}{\text{e}}^{\frac{-{\left(x-\mu \right)}^{2}}{2\sigma^{2}}}\log\frac{1}{\sqrt{2\pi \sigma^{2}}}{\text{e}}^{\frac{-{\left(x-\mu \right)}^{2}}{2\sigma^{2}}}\text{dx}\\ \;=\frac{1}{2}log(2\pi \text{e}\sigma^{2}), \end{array}$$

where, x_i_[n] is assumed to satisfy the Gaussian distribution of N(μ, σ^2^) (Shi et al., 2013).

Sliding window technology is applied for feature extraction in this study. The Hanning window with a length of 1 s and an increment of 1 s increment without overlap is taken to segment the EEG signal for feature extraction.

In order to remove the noise which has nothing to do with the emotional states, this paper uses Savitzky-Golay method with span of 5 and degree of 3 to smooth the data. Dimensionality reduction could reduce the computational burden and increase the stability of the computation (Duan et al., 2013). Moreover, it is a practical solution to avoid “dimension disaster” (Duan et al., 2013). Popular dimensionality reduction methods include principal component analysis (PCA), minimum-redundancy-maximum-correlation (MRMR), and so on (Peng et al., 2005). Although PCA can reduce the feature dimensions, it cannot preserve the original domain information, such as channel and frequency after the transformation. Hence, this paper chooses the MRMR algorithm to select a feature subset from an initial feature set (Zheng et al., 2017). The MRMR algorithm utilizes mutual information as the relevance measure with the max-dependency criterion and minimal redundancy criterion. The max-relevance criterion searches for features satisfying with the mean value of all the mutual information values between the individual feature x_i_ and class *c* as follows,

(4) $$\begin{array}{l} \max D\left(\text{S},\text{c}\right),\text{D}=\frac{1}{\left|\text{S}\right|}\underset{{\text{x}}_{\text{i}}\in \text{S}}{\sum }\text{I}\left({\text{x}}_{\text{i}};\text{c}\right) \end{array}$$

When two features are highly dependent on the same class, if one of the features is removed, the overall class distinction ability does not change much. The following minimal redundancy condition can thus be added to select for mutually exclusive features,

(5) $$\begin{array}{l} \min\left(\text{S}\right),\text{R}=\frac{1}{{\left|\text{S}\right|}^{2}}\underset{{\text{x}}_{\text{i}},{\text{x}}_{\text{j}}\in \text{S}}{\sum }\text{I}\left({\text{x}}_{\text{i}};{\text{x}}_{\text{j}}\right) \end{array}$$

The above two constraints are termed as “minimum-redundancy-maximum-correlation” (MRMR). We define the operator ∅(D, R) to combine D and R, and the simplest definition can be expressed as.

(6) $$\begin{array}{l} \max\emptyset (\text{D},\text{R)},\emptyset =\text{D}-\text{R} \end{array}$$

### 3.4. Multi-Feature Deep Forest Method

As an extension of random forest, deep forest is different from general random forest. Random forest is based on decision trees and uses the idea of ensemble learning to classify data. Deep forest adopts cascade structure which combines the characteristics of the neural network to further improve the recognition of random forest, and the cascade layer can automatically adjust the optimal number of classification layers (Xu et al., 2019). Deep forest automatically optimizes the structure of deep forest by comparing the classification performance of adjacent layers.

**Figure 3:**
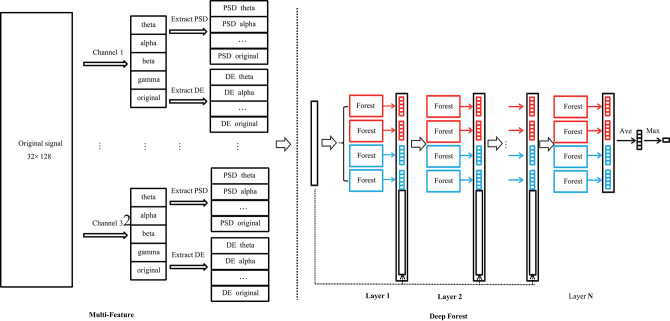
The structure of multi-feature deep forest. It includes a multi-feature extraction part (left panel) and a deep forest part (right panel). In multi-feature extraction stage, PSD and DE features are extracted from the EEG signal. In deep forest part, each layer of the cascade consists of two types of random forests with different colored blocks, and the number of layers is optimized during the training stage.

The structure of the proposed MFDF method is demonstrated in Figure 3. It includes two parts. One is multi-feature extraction, and the other is deep forest architecture. In the multi-feature extraction stage, PSD and DE features are extracted from different wave bands of EEG signals or original signals for each EEG channel. The size of the extracted feature is 320 ×1 (32 channels and 10 types of feature for each channel). The architecture of the deep forest can be found in the right panel of Figure 3. In each layer, four random forests are included. Two of random forest set the number of tree node's split feature by the number of square root for the total number of features, and rest two forests set it by the logarithm for the total number of features (Zhou and Feng, 2017; Yao et al., 2019; Cheng et al., 2020). A Iterative Dichotmizer 3 (ID3) decision tree is utilized in this study (Zhou and Feng, 2017; Yao et al., 2019; Cheng et al., 2020).

In the deep forest, the output vector of the current layer is taken as enhancement features that is further used to combine original data as the new input of the next layer, as seen in the right panel of Figure 3. The output vector is actually a vector of classification probability for each class. The classification probability is statistically calculated from the output of each decision tree. In the current study, five classes of emotions are investigated. Each layer would thus generate 20 enhancement features by four random forests. The number of layers is not fixed in this study, and it is determined by the algorithm via evaluate whether additional layer could improve the classification performance.

### 3.5. Confirmation of Hyper-Parameters of Deep Forest

**Figure 4:**
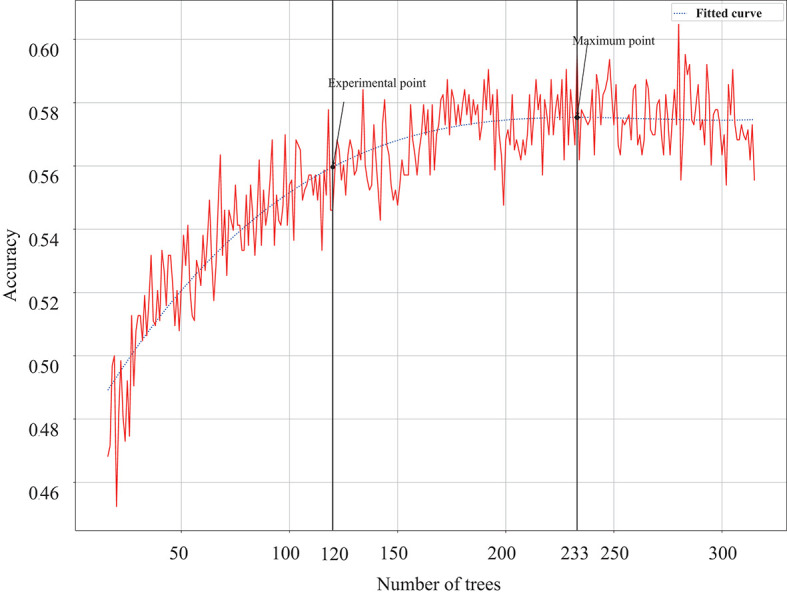
The change of classification accuracy along with the number of trees in each forest, and the highest accuracy point and the 97% turning point are marked on the fitted curve.

It is well-known that the number of trees of a random forest would influence its classification performance, and a large number of trees would increase computing burden. To determine the number of trees in a forest, subject's (S5) data are used to evaluate how the number of trees influence the classification accuracy. Figure 4 shows the accuracy change along the increase of the number of trees. It can be found that with the increase of tree number, the accuracy shows a clear improvement. When the number of trees increases to 200, accuracy enhancement becomes stable. In order to balance computing resources and classification accuracy, this study sets the number of trees in each random forest to 120 trees, which is the 97% turning point of the highest accuracy. Other parameters of random forest are set as follows. The maximum depth of decision tree is set to 14. The minimum number of samples required to split middle nodes is set to five. This paper also uses out-of-bag samples to estimate the generalization accuracy. To tackle the problem of data imbalance, the samples in a small group would be more frequently chosen during the training stage.

## 4. Experimental Result

**Figure 5:**
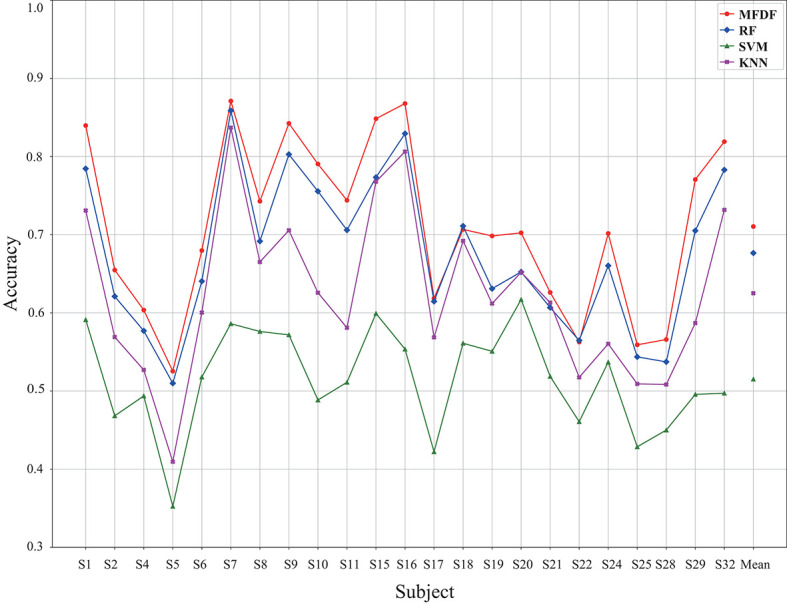
The classification accuracy across different subjects under the input of PSD and DE feature without dimension reduction, in which the classifiers of MFDF, RF, SVM, and KNN are compared.

The proposed MFDF is compared with three traditional classifiers (i.e., RF, SVM, and KNN), and 5-fold cross-validation is applied to obtain the classification accuracy. For SVM classifier, linear kernel is applied with the penalty coefficient at 0.8. The K coefficient is set to 5 for KNN classifier. In the random forest, 100 decision trees are included, and ID3 algorithm is used for training. The average accuracy and the accuracy for different subjects are demonstrated in Figure 5. It can be found that the average accuracy is around 71, 68, 52, and 63% for MFDF, RF, SVM, and kNN, respectively. The average recognition accuracy of the proposed model is 19.53% higher than SVM, 3.4% higher than RF, and 8.54% higher than KNN in Table 2. Besides, the proposed MFDF attains the highest accuracy up to 86% for subject s7 and s16.

**Table 2:** A comparison of classification accuracy by four classifiers with original feature as input.

	**Highest recognition** **accuracy (%)**	**Lowest recognition** **accuracy (%)**	**Average recognition** **accuracy (%)**
MFDF	87.90	52.54	71.05 ± 10.61
RF	85.87	51.00	67.65 ± 9.72
SVM	61.71	35.23	51.52 ± 6.41
KNN	83.69	40.96	62.51 ± 10.30

**Figure 6:**
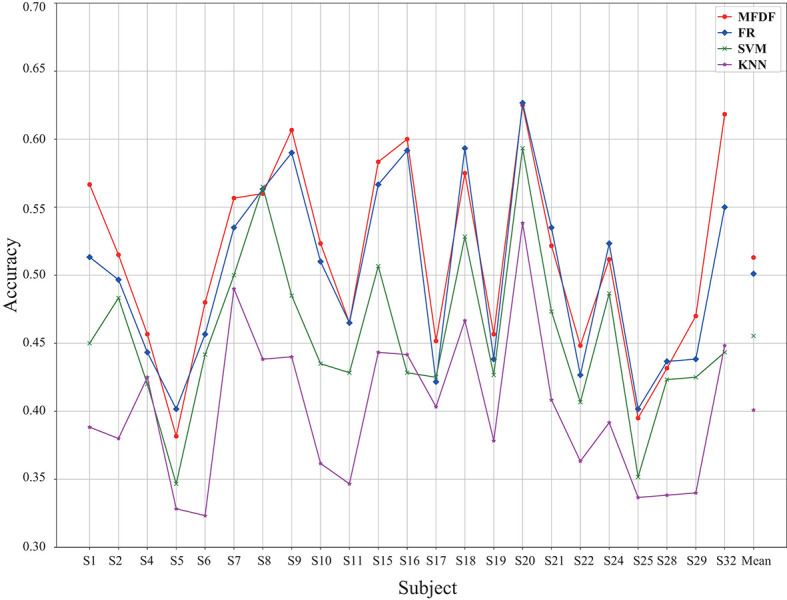
The classification accuracy across different subjects under the input of PSD and DE feature with dimension reduction across different subjects, in which the classifiers of MFDF, RF, SVM, and KNN are compared.

The reliability and effectiveness of the MFDF are also evaluated through investigating different types of data as input (i.e., raw data, features, and features after dimension reduction). The experimental results with dimension reduced feature as input are demonstrated in Figure 6. The average recognition accuracy of the proposed method is 51.30%. Although it is much lower than that without dimensionality reduction, this accuracy is still higher than that of the compared classifiers. Table 3 shows that the average recognition rate of the model is 5.76% higher than SVM, 1.19% higher than RF, and 11.21% higher than KNN.

**Table 3:** A comparison of classification accuracy by four classifiers with dimension reduced feature as input.

	**Highest recognition accuracy (%)**	**Lowest recognition accuracy (%)**	**Average recognition accuracy (%)**
MFDF	62.50	38.17	51.30 ± 7.02
RF	62.67	40.17	50.11 ± 6.73
SVM	59.33	34.67	45.54 ± 5.75
KNN	53.83	32.33	40.09 ± 5.54

**Figure 7:**
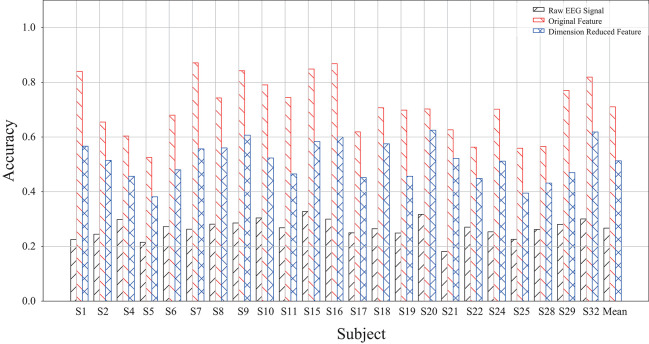
A comparison of MFDF with different input of raw EEG signal, original features, and dimension reduced features.

This study also takes raw data without feature extraction as the input for deep forest and investigates the accuracy. The results are demonstrated in Figure 7. The average accuracy without dimensionality reduction processing is 71.05%, and the average accuracy for dimensionality reduction processing is 51.30%. The average accuracy of the original data is 26.71%. It can be found that using original feature as input obtains the highest classification accuracy, which indicates that deep forest can deal with features rather than original data more successfully in the current case study.

In addition, for real-time application, the computing cost is very important. Table 4 demonstrates the computational cost for feature extraction with or without dimension reduction, and the corresponding accuracy of subject S1. It can be found that feature extraction takes a large amount of time, which is much longer than the time cost of classification. The total time of MFDF, SVM, KNN, and RF for feature extraction and classification of a sample is 38, 25.56, 25.22, and 25.65 ms, respectively. Although MFDF takes more time than the other three traditional classifiers, it achieves the relative higher classification accuracy.

**Table 4:** The computational cost for feature extraction and classification per sample.

	**Features extraction (ms)**	**Classification (ms)**	**Accuracy (%)**
MFDF(1)	25	13	71.05
MFDF(2)	44	11	51.30
SVM	25	0.56	51.52
RF	25	0.65	67.65
KNN	25	0.22	62.51

The reported accuracy is from subject S1. MFDF(1) indicates the results without dimensionality reduction, and MFDF(2) is the ones after dimensionality reduction. The compared SVM, RF, and kNN classifier takes original feature as input.

## 5. Discussion

EEG-based emotion recognition is a hot-spot in recent years. Many researchers have proposed effective classification models to improve emotion recognition accuracy (Huang et al., 2019; Yao et al., 2019). The extraction of distinguish and consistent EEG features are critical for classification systems. PSD and DE are two classic features set for EEG-based emotion recognition (Naderi and Mahdavi-Nasab, 2010; Duan et al., 2013; Shi et al., 2013), which are selected as the input feature for the proposed MFDF method. This study also finds that the implementation of dimensionality reduction would negatively influence the classification accuracy, which is inconsistent with the results obtained by Zheng et al. (2017) who find that dimensionality reduction does not affect the performance of our model greatly. Additionally, it is also found that deep forest cannot achieve acceptable classification accuracy with raw EEG signal as input, although recent studies show a tendency to use original data as input for deep forest. For instance, Cheng et al. (2020) utilized the raw multi-channel EEG data as 2D input and achieved more than 97% classification for a two-class classification problem of the state of valence and arousal; it is not clear, however, whether the extraction of feature can further improve the classification accuracy. In sum, the result of the current study indicates that it is rational to use PSD and DE feature as input for deep forest.

**Figure 8:**
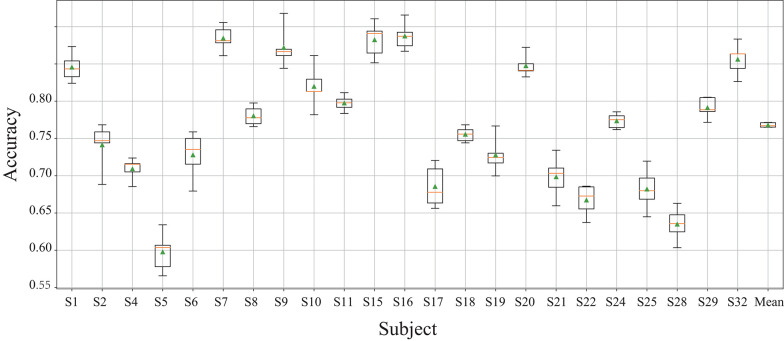
The accuracy for the classification of 4 types of emotions by MFDF. The error bar is obtained by 5-fold cross validation.

Table 5 lists related studies that used the DEAP dataset for pattern recognition. The baseline accuracy for DEAP is only about 60% for two-class classification problems, and this was published together with DEAP by Koelstra et al. (2011). In the two-class classification problem, Yang et al. (2018a) obtained the accuracy of 91% via the parallel convolutional recurrent neural network, which is the highest classification accuracy within these studies on two-class classification. For three-class emotion classification, Yao et al. (2019) used deep forest with multi-scale window (MSWDF) to identify the emotions of pleasure, relaxation and sadness and achieved an accuracy of 84.90%. The proposed method achieved an accuracy of 76.8% for four emotions (shown in Figure 8, which is higher than the ensemble convolutional neural network (ECNN) approach that obtained the accuracy of 73.76% (Huang et al., 2019). Besides, the current study takes five emotions for classification, including a neutral emotion, and achieved an average accuracy of 71.05%.

**Table 5:** The reported accuracy by the literatures with DEAP database.

**Study**	**Results**
Koelstra et al. (2011)	62.0, 57.6% for valence and arousal (two-classes) with all 32 participants.
Xie et al. (2018)	79.06 and 77.19% for valence and arousal (two-classes) with all 32 participants.
Yang et al. (2018a)	90.80 and 91.03% for valence and arousal (two-classes) with all 32 participants.
Chung and Yoon (2012)	66.6, 66.4% for valence and arousal (two-classes), 53.4, 51.0% for valence and arousal (three-classes) with all 32 participants.
Yao et al. (2019)	84.90% for pleasure, relax, sadness (three-classes)with all 32 participants
Liu and Sourina (2013)	63.04% for arousal-dominance recognition (four-classes) with the selected 10 participants.
Zheng et al. (2017)	69.67% for quadrants of VA space (four-classes) with all 32 participants.
Huang et al. (2019)	73.76% for relax, depression, excitement, fear (four-classes) with all 32 participants
Our method	71.05% for angry, happy, sad, pleasant, and neutral (five-classes) with the selected 23 participants

## 6. Conclusion

This paper proposes an emotion recognition method based on gcForest (MFDF) for emotion recognition, which takes human-crafted features (i.e., PSD and DE) as the input for deep forest. The proposed methods demonstrate a competitive performance (71% accuracy for five types of emotions) in the comparison with traditional classifiers. The results indicate that using feature as input can obtain much higher accuracy than using raw EEG signals. In the future, deep forest will be further optimized through the combination of raw EEG data together with EEG features as input for deep forest. The issue of cross-subject emotion recognition will be investigated to establish a more general classification model.

## Data Availability Statement

The original contributions presented in the study are included in the article/supplementary material, further inquiries can be directed to the corresponding author/s.

## Ethics Statement

Ethical review and approval was not required for the study on human participants in accordance with the local legislation and institutional requirements. The patients/participants provided their written informed consent to participate in this study.

## Author Contributions

YF: original motivation and idea and language revising. HY: coding and drafted manuscript. XZ: project supervisor. HL: method of deep forest algorithm. BT: final check. All authors contributed to the article and approved the submitted version.

## Conflict of Interest

The authors declare that the research was conducted in the absence of any commercial or financial relationships that could be construed as a potential conflict of interest.
